# Ultraviolet Light, Hydrogen Peroxide, Peracetic Acid, and Ozone as Sanitizers for Hatching Eggs: A Review

**DOI:** 10.3390/ani16132108

**Published:** 2026-07-07

**Authors:** Gabriel da Silva Oliveira, Igor Rafael Ribeiro Vale, Concepta McManus, Vinícius Machado dos Santos

**Affiliations:** 1 Faculty of Veterinary Medicine and Animal Science, University of São Paulo, São Paulo 05508-270, Brazil; 2 Faculty of Agronomy and Veterinary Medicine, University of Brasília, Brasília 70910-900, Brazil; 3 Laboratory of Poultry Science, Federal Institute of Brasília—Campus Planaltina, Brasília 73380-900, Brazil; 4 Center for Nuclear Energy in Agriculture (CENA), University of São Paulo, São Paulo 13416-000, Brazil

**Keywords:** bacterial contamination, egg sanitization, eggshell, poultry health, poultry production, poultry sanitizers, synthetic products

## Abstract

Sanitizing hatching eggs has become increasingly recommended as a strategy to reduce infections, limit the spread of bacteria, and promote healthier conditions for developing embryos. The absence of this procedure can contribute to lower hatchability rates in commercial hatcheries. Therefore, investigating different sanitizers applied to hatching eggs is essential. This review synthesized evidence from Google Scholar up to June 2026, examining the effects of ultraviolet light (UV), hydrogen peroxide (H_2_O_2_), peracetic acid (PAA), and ozone (O_3_) on eggshell bacterial load, eggshell structural integrity, and hatchability. The findings reveal that these sanitizers have the potential to reduce eggshell bacterial load without apparently causing significant structural alterations or compromising hatchability. However, it also became clear that outcomes depend strongly on the protocol configuration, as adverse effects may occur under different conditions, such as concentration, exposure time, or application method. Overall, UV, H_2_O_2_, PAA, and O_3_ can be used to sanitize hatching eggs, if protocols prioritize microbiological safety without impairing embryonic performance, thereby avoiding negative impacts on hatchability rates.

## Simple Summary

Ensuring microbiological safety throughout the entire production process is one of the main ongoing challenges in the poultry industry. One of the limitations on poultry productivity is poultry mortality caused by microorganisms such as bacteria. Eggs are an important source of bacterial contamination. In this context, sanitizers act as key agents in bacterial control programs. This review synthesized evidence on the use of ultraviolet light (UV), hydrogen peroxide (H_2_O_2_), peracetic acid (PAA), and ozone (O_3_) for egg sanitization, with emphasis on their effects on eggshell bacterial load, eggshell structural integrity, and hatchability.

## 1. Introduction

**Figure 1:**
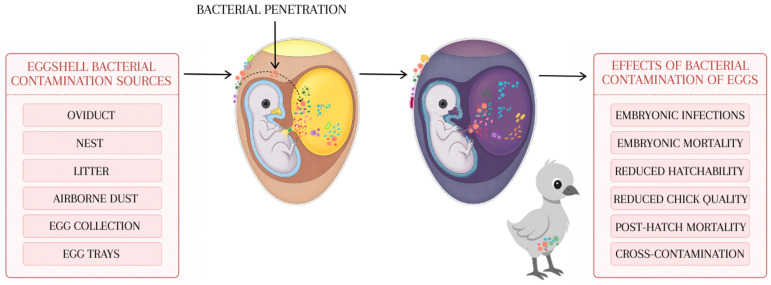
Proposed pathway linking eggshell bacterial contamination originating from poultry production environments to adverse outcomes during incubation and the post-hatch period.

Bacterial contamination of hatching eggshells is recognized not only as a factor associated with embryonic mortality and reduced hatchability, but also as an important source of cross-transmission and compromised sanitary quality of chicks after hatching. This contamination is widely documented in poultry production, with studies reporting high total bacterial loads and the presence of *Staphylococcus* spp., Enterobacteriaceae, coliforms, and *Salmonella* spp. on the egg surface [1,2]. Evidence indicates that the presence of these microorganisms is directly associated with embryonic infections, a key factor contributing to embryonic mortality and reduced hatchery and farm performance [3]. Figure 1 illustrates the main sources of eggshell bacterial contamination, subsequent bacterial penetration through the eggshell, and the potential consequences for embryonic development and post-hatch performance. Therefore, bacterial contamination of hatching eggs remains a major concern in poultry production systems, despite significant advances in management practices, biosecurity barriers, and containment measures.

The bacterial control of the eggshell surface after collection using ultraviolet light (UV), hydrogen peroxide (H_2_O_2_), peracetic acid (PAA), and ozone (O_3_) has been proposed and suggested [4,5,6,7,8,9,10,11,12]. Understanding why these compounds have been proposed is essential for improving sanitization protocols and guiding professionals who wish to apply them safely and effectively to control losses from bacterial contamination of eggs. Studies typically assess the performance of sanitizers using parameters such as bacterial counts on the eggshell, which indicate the number of viable bacteria that may pose risks to the embryo, hatchability, which represents the proportion of fertile eggs that successfully produce chicks, and other aspects related to eggshell structure and poultry. Some reviews on sanitizing hatching eggs have addressed alternatives to formaldehyde. However, no review published in the last decade has provided a detailed, comparative discussion of the use of UV, H_2_O_2_, PAA, and O_3_ for hatching-egg sanitization, particularly regarding their effects on these factors. Therefore, this review aimed to synthesize the available findings on the effects of UV, H_2_O_2_, PAA, and O_3_ on eggshell bacterial load, eggshell structure, and hatchability.

## 2. Methods

A preliminary bibliographic search was conducted in Google Scholar on 5 October 2025, to identify the most appropriate keywords for the advanced search in the same database. After this step, the following keywords, in both English and Portuguese, were used in the advanced search: ultraviolet light, hydrogen peroxide, peracetic acid, ozone, UV, H_2_O_2_, PAA, O_3_, sanitization, sanitizers, disinfection, disinfectants, treatments, antibacterials, formaldehyde, eggshells, eggshell damage, eggshell structure, eggshell protein matrix, eggshell ultrastructure, eggshell quality, egg proteins, table eggs, hatching eggs, hatchability, embryo, poultry farm, and hatchery, employing the Boolean operators AND and OR. The search retrieved records based on the occurrence of these terms anywhere in the documents. The search strategy was last updated on 17 June 2026.

Research articles, conference papers, reviews, books, and book chapters published in English or Portuguese that addressed at least one of the predefined topics related to bacterial contamination of the eggshell, egg sanitization using UV, H_2_O_2_, PAA, or O_3_, and their effects on eggshell bacterial counts, eggshell structure, and hatchability were included with no restrictions on publication date. However, studies specifically focused on sanitizing eggs using UV, H_2_O_2_, PAA, or O_3_ were considered only if published from 2015 onward. An exception was made for studies addressing the effects of these sanitizers on eggshell structure, for which earlier publications were also considered to support the discussion. For studies focused on UV, only those evaluating UV-C wavelengths (200–280 nm) were considered, as this spectral range, unlike UV-A and UV-B, exhibits high antibacterial efficacy and is the form of UV most widely investigated and applied for egg sanitization. Therefore, whenever the term UV is used throughout this review, it refers exclusively to UV-C radiation unless otherwise specified.

Studies that assessed the use of sanitizers on table eggs were also considered to support the discussion on bacterial reduction and eggshell structure. The management of table eggs generally differs from that of hatching eggs within production systems. However, the inclusion of studies conducted on table eggs is justified by the structural similarity of the eggshell, which presents a comparable composition and physical organization regardless of its intended purpose. Furthermore, this discussion is not focused on differences in microbial load resulting from management practices, environmental conditions, or production systems, but rather on the direct effects of sanitizers on microorganisms present on the eggshell surface. Therefore, the mechanisms of interaction between sanitizers, the eggshell surface, and the microorganisms present on it are likely to be similar, allowing for a joint discussion of their effects.

The reference lists of the retrieved materials were also manually screened to identify additional relevant sources. The search process for each topic was concluded when sufficient information was obtained to adequately support and structure the proposed discussion. All selected materials were imported into Mendeley for organization and further review. The titles and abstracts of documents that potentially met the search criteria were assessed, and the selected materials were read in full. Materials that did not address at least one of the defined topics, were not available in Portuguese or English, or no longer contributed to the development of the topic after its main idea had been consolidated, were excluded.

## 3. Bacterial Contamination of Eggs

Under natural conditions, eggshell contamination does not originate solely from the hens themselves but also from the surfaces with which the eggs come into contact immediately after laying, such as litter. This material can significantly influence the bacterial composition of contamination [13]. The eggshell, therefore, can act as an essential vehicle for sanitary relevance. In an analysis conducted on eggs collected from poultry farms, the following bacteria were identified on the eggshell: *Alcaligenes faecalis*, *Bacillus cereus*, *Bacillus megaterium*, *Bacillus polymyxa*, *Escherichia coli*, *Klebsiella oxytoca*, *Pseudomonas aeruginosa*, *Salmonella* Typhimurium, *Staphylococcus aureus*, and *Staphylococcus epidermidis* [14]. Another study isolated *Escherichia coli*, *Salmonella* spp., *Shigella* spp., *Staphylococcus* spp., and *Pseudomonas* spp. from eggshells obtained from different poultry farms [15].

Previously, it was reported that the bacterial flora of embryos that died before hatching included members of the Enterobacteriaceae family, particularly *Escherichia coli*, *Proteus* spp., *Salmonella* spp., *Citrobacter* spp., and *Klebsiella* spp. The authors suggested that this contamination was associated with eggshell contamination, given that these bacteria can penetrate the shell and contaminate the embryo [16]. Oliveira et al. [17] reviewed the factors associated with bacterial penetration and observed that this process is associated with intrinsic egg characteristics as well as extrinsic factors related to egg handling, including the absence or incomplete deposition of the cuticle, eggshell pore diameter, exposure of eggs to temperature fluctuation regimes, eggshell dynamic stiffness, high levels of surface contamination, and the motility and non-clustering properties of certain bacteria. Therefore, sanitizing the eggshell surface after laying is essential to minimize embryonic infections and reduce incubation-related losses.

## 4. Sanitization of Hatching Eggs

**Figure 2:**
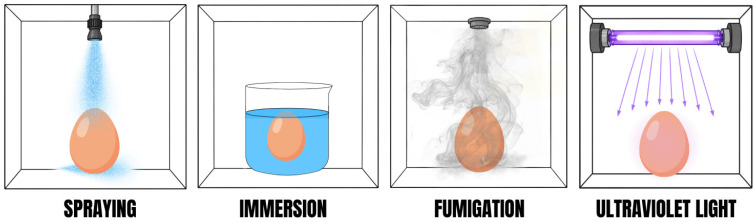
Main methods for sanitizing hatching eggs.

The sanitization of hatching eggs involves applying a synthetic chemical compound to the eggshell surface immediately after collection, in an appropriate environment, and in accordance with strict sanitary and safety protocols. However, plant-derived compounds have also been applied as sanitizing agents for this purpose at both laboratory and commercial levels [2,18,19,20]. Both nest- and floor-collected eggs should undergo sanitization. The effectiveness of sanitization depends on the application method [21]. The most used and tested methods for applying these compounds to eggs are spraying, immersion, and fumigation, although UV has also been well-tested (Figure 2) [22,23]. Each method has its own characteristics that may be beneficial or detrimental to the process (Table 1). Therefore, planning before sanitization is the best approach to identify the most appropriate method for applying sanitizers and to minimize their limitations.

## 5. Formaldehyde as a Conventional Sanitizer for Hatching Eggs and Its Limitations

Formaldehyde is a broad-spectrum sanitizer that has been used in the poultry industry for many decades, mainly for bacterial control in eggs before and during incubation. It inhibits bacterial growth by disrupting protein structure and interacting with purine bases in DNA and RNA, ultimately resulting in bacterial death [26,27]. The expansion of the poultry industry and the increase in egg production intensified the need for practical, efficient, large-scale sanitary control methods, thereby promoting the widespread use of formaldehyde on poultry farms and in commercial hatcheries. Although formaldehyde fumigation aims to protect poultry production from a sanitary standpoint and ensure productivity, its extremely hazardous handling poses significant health risks, particularly under inadequate handling and application conditions [28]. For example, its use may cause respiratory and brain damage in poultry embryos [19,29] and cancer in humans [30]. Therefore, other products have been tested to reduce formaldehyde use without compromising poultry productivity.

## 6. Sanitizing Hatching Eggs Using Ultraviolet Light (UV), Hydrogen Peroxide (H_2_O_2_), Peracetic Acid (PAA), and Ozone (O_3_)

UV, H_2_O_2_, PAA, and O_3_ have also been used as poultry sanitizers for treating hatching eggs. UV in the 200–280 nm range of the electromagnetic spectrum represents the wavelength most used for antibacterial treatment [31], including for egg treatment [25]. H_2_O_2_ is a chemical compound composed of two hydrogen atoms and two oxygen atoms, commercially supplied as aqueous solutions in which the stated concentration reflects the percentage of H_2_O_2_ by weight [32]. PAA is a colorless solution consisting of a monoacetylated derivative of H_2_O_2_ and is commercially formulated as a mixture of PAA, acetic acid, H_2_O_2_, and water [33]. O_3_, a triatomic molecule and an allotropic form of oxygen (O_2_), can be generated by electrochemical processes, UV irradiation, corona discharge, or radiochemical methods [34].

Antibacterial action is a minimum requirement for a product to be considered suitable for application to hatching eggs. *Salmonella* spp. and *Escherichia coli* are among the bacteria susceptible to sanitization protocols involving UV, H_2_O_2_, PAA, and O_3_ (Table 2) and, as mentioned earlier, can be detected on eggshells and embryos, where they may pose risks to embryo survival. However, it is important to clarify that the susceptibility observed under in vitro conditions does not necessarily translate to the same level of efficacy when sanitizers are applied to eggshells. The antibacterial activity of these compounds arises from their absorption of UV light at 254 nm, which damages bacterial DNA, compromising replication and transcription and thereby interrupting the cell cycle and leading to bacterial death [35,36]. When bacteria are exposed to H_2_O_2_ or O_3_, an intense intracellular oxidative stress occurs, capable of damaging lipids, proteins, and DNA, thereby compromising bacterial survival [37,38]. Regarding PAA, its antibacterial mechanism may not directly involve DNA damage but is likely related to the damage it causes to membrane proteins [39].

### 6.1. Effects of Sanitizers on Bacterial Control of Eggshells

Branco et al. [57] evaluated the effectiveness of UV in reducing the bacterial load on the eggshell surface of hatching eggs, comparing exposure times (5–9 min) using a 254 nm UV lamp at an effective intensity of 0.23 mW/cm^2^. The results showed that all exposure times significantly reduced the bacterial load compared with the non-sanitized group, with greater efficiency as exposure time increased. The control treatment presented 3.26 log_10_ CFU/egg, while the values decreased to 2.58 log_10_ (5 min), 2.25 log_10_ (7 min), and 2.00 log_10_ CFU/egg (9 min) with varying exposure times. Mousa-Balabel et al. [58] reported that sanitizing hatching eggs with 1.4% H_2_O_2_ significantly reduced the total bacterial load on the eggshell, achieving an 84.08% reduction. Al-Ajeeli et al. [12] observed that spraying PAA at 135 ppm significantly reduced the mesophilic aerobic bacterial counts on the eggshell surface. Immediately after application (day 0), the bacterial load decreased from 3.17 to 2.60 log_10_ CFU/egg. After 7 days of application, it further declined to 1.83 log_10_ CFU/egg. Although a slight increase was recorded after 14 days (2.08 log_10_ CFU/egg), the values remained significantly lower than those of the control group across all evaluated periods. Koc and Aygun [59] evaluated O_3_ concentrations ranging from 1 to 7% applied to hatching eggs and found that only the 7% treatment significantly reduced total aerobic mesophilic bacteria throughout the incubation period. On the first day, the count of 2.89 log_10_ CFU/egg observed in the control group declined to 1.21 log_10_ CFU/egg in the 7% O_3_ group. This level of effectiveness persisted on day 7, when the treated eggs showed 1.28 log_10_ CFU/egg, compared with 3.04 log_10_ CFU/egg in the control group. By day 14, the difference persisted, with 1.48 log_10_ CFU/egg in the treated eggs and 3.07 log_10_ CFU/egg in the control group.

Other investigations have evaluated the effectiveness of UV, H_2_O_2_, PAA, and O_3_ in reducing eggshell bacterial load in both hatching and table eggs, as summarized in Table 3 and Table 4. Studies that tested UV show a considerable consistency in their results. Even with the heterogeneity among protocols, whether in exposure time or applied intensity, the evidence indicates substantial reductions in bacterial load. H_2_O_2_ and PAA, applied mainly by spraying, and O_3_, more frequently used through fumigation, also demonstrate a relevant ability to reduce eggshell bacterial contamination. However, these compounds show greater sensitivity to protocol variables such as concentration and exposure time. The results for all sanitizers were not limited to reductions in bacterial load (Table 3 and Table 4). In some studies, the observed effects were not significant, whereas in others, bacterial counts increased significantly. Therefore, all four sanitizers can be effective in reducing bacterial contamination on the eggshell surface, but their efficacy depends on the application plan adopted.

### 6.2. Effects of Sanitizers on Eggshell Structure

In addition to the antibacterial potential of sanitizers in controlling bacterial contamination on the eggshell, it is essential to understand how, and to what extent, these sanitizers may pose risks to the eggshell structure. The eggshell plays a well-established role as a primary support for proper embryonic development. In addition to its various functions, it acts as a natural barrier to bacterial penetration [86] and provides the necessary substrate for the calcification of the embryonic skeletal system [87]. For this reason, understanding how UV, H_2_O_2_, PAA, and O_3_ treatments interact with the eggshell is essential for designing protocols that preserve its structural integrity and, consequently, prevent significant negative impacts on the embryo in microbial terms and skeletal formation.

Avcilar and Yilmaz [88] reported that the application of UV at 254 nm for 3 min on eggs did not cause significant changes in shell-breaking strength or shell weight. However, the authors observed a significant reduction in shell thickness, suggesting that this decrease may be related to UV-induced damage to the cuticular layer on the shell surface. Nevertheless, Coulibaly et al. [62] evaluated egg sanitization using UV at 254 nm for 1 min and found no significant effects on eggshell characteristics, including overall shell thickness, the structure of the mammillary, palisade, and vertical layers (both thickness and relative proportion), and the morphology and arrangement of cones. Similarly, Holck et al. [70] used scanning electron microscopy and observed that eggs exposed to UV at 10 mW/cm^2^ for 1 min showed no visible alterations in the protein cuticle. In addition, Panini [83] observed that the eggshell color changed when eggs were exposed to UV for 10 min, suggesting that lower exposure levels are recommended to prevent alterations in shell pigmentation.

Spraying with 1.56% H_2_O_2_ did not affect the thickness or strength of hatching eggshells [61], a result similar to that observed by Melo et al. [22] when using a 3% concentration. However, after the eggs were immersed in a 5% H_2_O_2_ solution, Bekhet [89] observed a reduction in eggshell thickness. At the microscopic level, eggshell membranes immersed in a 35% H_2_O_2_ solution for 4–24 h exhibited structural alterations, characterized by changes in pore size, fiber cross-linking density, and surface topography, proportional to the exposure time [90]. Sheldon and Brake [91] reported that, despite the oxidative potential of H_2_O_2_ and the possibility of inducing effects on the eggshell cuticle, eggshell permeability was not affected after spraying a 5% H_2_O_2_ solution. However, at the same concentration but using immersion as the application method, Zeweil et al. [92] observed that H_2_O_2_ affected eggshell permeability.

The application of PAA at concentrations between 75 and 300 ppm, or in the commercial 15% formulation, caused evident structural alterations to the eggshell, including microfissures and microfragments in the cuticle, thereby increasing pore exposure [93]. Similarly, the spraying of PAA at a concentration of 355 ppm significantly reduced the thickness of the calcified region of the eggshell. According to the study, PAA also reduced the palisade and mammillary layers, although the effect was not statistically significant. This tendency toward thinning in these inner layers may help explain the overall decrease in total shell thickness observed after the sanitizing agent was applied [80]. However, alterations in shell thickness or strength were not observed by Melo et al. [22] after spraying a 0.3% PAA solution.

Soares et al. [94] reported that the extent of eggshell damage following O_3_ exposure was directly related to the degree of exposure. According to the authors, treating eggs with 60 ppm O_3_ for 120 min resulted in the formation of additional microfissures, while the pores remained intact and protected by the cuticle. After 300 min, however, these fissures became more extensive, and some regions began to exhibit exposed pores. Fuhrmann et al. [95] observed that applying O_3_ at 10 mL/L for 20 min destroyed cuticle proteins. Clímaco et al. [61] reported that eggshell thickness and shell strength, measured after sanitization with O_3_ gas at concentrations of 5–10 ppm for 20 min, did not undergo any detectable changes. Conversely, Yüceer et al. [96] reported that the breaking strength and puncture resistance of eggshells exposed to O_3_ at 2, 4, or 6 ppm were significantly reduced as both O_3_ concentration and exposure time (2 or 5 min) increased.

Based on the studies reviewed above, UV, H_2_O_2_, PAA, and O_3_ can, depending on the protocol used, trigger significant alterations in the eggshell’s structural integrity. Although macrostructural modifications, such as detectable variations in shell thickness, are not always evident, microstructural disturbances may occur silently and pose substantial risks to the mechanical stability of the egg. As the concentration of the sanitizer or the duration of exposure increases, progressively greater levels of shell damage are observed, ranging from subtle microfissures to more evident deterioration.

One possible explanation for the eggshell damage observed in some UV, H_2_O_2_, PAA, and O_3_ treatment protocols is the alteration of the eggshell protein matrix [90,95,97], which represents approximately 2% of the calcified eggshell and is composed of proteins, glycoproteins, phosphoproteins, and proteoglycans [98]. UV may induce protein photo-oxidation, resulting in protein fragmentation and aggregation [97]. H_2_O_2_ may promote structural modifications in proteins through the formation of disulfide bonds resulting from oxidative processes [90]. The action of PAA may be linked to the presence of H_2_O_2_ in its formulation, thereby promoting similar oxidative processes. O_3_ may promote the oxidation of sulfhydryl groups and extensive alterations in the three-dimensional structure of proteins [95,97]. These effects may compromise the integrity of the eggshell protein matrix, increasing its permeability, favoring microbial penetration, and dysregulating water loss during incubation. As a result, possible deleterious effects on hatchability and post-hatch survival may occur. Therefore, future studies must adopt standardized protocols that concomitantly integrate macro- and microstructural assessments to identify sanitization conditions that meet microbiological efficacy criteria without significantly compromising eggshell integrity at any structural level.

### 6.3. Effects of Sanitizers on Hatchability

Events that occur throughout embryonic development, mainly when triggered by external factors such as sanitizers, decisively influence hatchability rates. This productive indicator, measured only at the end of incubation, functions as a summary of what occurred with the embryo throughout development. It therefore reflects the accumulated effects that sanitizers may exert during this period. These effects can include alterations in the eggshell, interference with adequate egg weight loss, changes in embryonic body weight, levels of embryonic contamination, variations in blood biochemical parameters, differences in the physical quality of the embryo, and levels of absorption of essential nutrients. Therefore, hatchability is a criterion used to evaluate the efficiency of a poultry sanitizer for hatching eggs [3].

Al-Shammari et al. [99] evaluated a UV sanitization protocol at 262 nm, applied for 30 min at 10 mW/cm^2^, and found that the treatment did not significantly affect hatchability. Wang et al. [100] found that using 3% H_2_O_2_ by spraying did not affect hatchability compared with untreated eggs, but hatchability was significantly reduced when the product was sprayed at 6% H_2_O_2_. Melo et al. [22] evaluated spraying hatching eggs with 0.3% PAA and reported that hatchability rates did not differ significantly from those of the control treatments. In the study by Souza et al. [101], the hatchability of eggs treated with O_3_ at the evaluated concentrations of 1.6 and 3.2 mg/mL, via 60 min fumigation, was significantly higher than that of untreated eggs. Taken together, the available evidence indicates that hatchability may remain unchanged, decrease, or even increase depending on the sanitization protocol used (UV, H_2_O_2_, PAA, or O_3_; Table 5). These findings are important because hatchability provides an initial indication of the biological safety of the sanitization procedure. Changes in this parameter may reflect direct toxic effects on the embryo or indirect effects resulting from alterations in the structure and functions of the eggshell, thereby compromising the conditions necessary for normal embryonic development.

However, hatchability alone does not allow for a complete understanding of the mechanisms responsible for these effects. Damage to eggshell structure is one of the hypotheses frequently discussed in the literature, as previously described. Therefore, to gain a deeper understanding of the effects of sanitization protocols on embryonic development and poultry quality, additional analyses have been conducted in studies evaluating UV light, H_2_O_2_, PAA, and O_3_ (Table 6). In addition to hatchability, parameters such as embryonic mortality at different incubation stages, chick quality, hatch weight, organ development, physiological alterations, yolk sac bacterial colonization, post-hatch performance, and histopathological evaluations have been used to assess the potential effects of these sanitizers. As observed for hatchability, these compounds may have no significant effect on these parameters, but may also exert beneficial or detrimental effects depending on the application protocol adopted (Table 6). Therefore, although hatchability is the flagship parameter in evaluating the safety of sanitization protocols, a combined assessment of multiple parameters can provide a broader understanding of these compounds’ effects on poultry and support the selection of the most appropriate protocol for each sanitizer.

It should be noted that the importance of sanitizing hatching eggs extends beyond hatchability and other incubation performance parameters, such as embryonic weight, chick weight at hatch, and chick quality, which are not always affected by sanitization protocols that employ UV light, H_2_O_2_, PAA, or O_3_. Its main objective is to provide a microbiologically safe environment for embryonic development by reducing the risk of contamination by potentially pathogenic bacteria. Embryos may develop normally and hatch without changes in hatchability, weight, or quality, even under conditions of bacterial contamination. However, this does not prevent them from carrying microorganisms that can compromise chick quality, post-hatch performance, flock health, and the biosecurity of production systems. Therefore, sanitization should be understood as part of a broader strategy to improve microbiological quality, protect embryonic health, and prevent the spread of bacteria throughout the poultry production chain.

### 6.4. Comparison of Sanitization Protocols Using UV, H_2_O_2_, PAA, and O_3_ with Formaldehyde

The comparison between protocols involving alternative sanitizing agents and those employing the reference sanitizer is necessary to determine the viability of these compounds for the intended purpose, thereby supporting their use and expanding the range of options available for hatching egg sanitization. Table 7 demonstrates that UV, H_2_O_2_, PAA, and O_3_ can reduce bacterial load on the eggshell and yield hatching results comparable to those obtained with formaldehyde. However, few comparative studies in the literature involve all these products, especially recent ones. These results suggest that protocols involving other products and methods may present productive potential similar to that of formaldehyde. As the effects of sanitizers on microbiological and productive parameters depend on several factors, such as concentration, application timing, exposure time, and product characteristics [108], it is essential to adopt previously tested protocols that provide results comparable to or superior to those obtained with formaldehyde, especially those using lower concentrations while maintaining similar efficacy. Such protocols are especially important for reducing the risk of potential toxic effects that may negatively affect hatchability.

## 7. Conclusions

In summary, the findings of this review indicate that UV, H_2_O_2_, PAA, and O_3_ can reduce bacterial contamination on the eggshell surface without necessarily compromising eggshell integrity, hatchability, or other incubation-related parameters. However, substantial variability was observed among the evaluated application protocols, and deleterious effects have been reported for all four sanitizers. These findings demonstrate that both antibacterial efficacy and embryonic safety are highly dependent on the application protocol employed. Therefore, the available evidence highlights the urgent need for standardized sanitization protocols that simultaneously ensure antibacterial efficacy, preserve eggshell structural integrity, and prevent adverse effects on embryonic development and hatchability. Such standardization should be supported by further investigations that address not only the traditional outcomes reviewed here but also embryonic bacterial load, detailed histological evaluations, and molecular-level studies of embryonic responses to these sanitizing agents, which remain limited in the current literature.

## Figures and Tables

**Table 1:** Main characteristics of the methods used to apply sanitizers to eggshell surfaces ^1^.

Method	Definition	Advantages	Disadvantages
Spraying	→ Dispersion of the sanitizing liquid mist using manual, semiautomatic, or automatic sprayers.	→ Control of the applied volume. → Control of the sanitizer concentration. → Does not require closed chambers.	→ Risk of incomplete surface wetting. → Increased difficulty in mass sanitization. → Risk of inducing thermal shock.
Immersion	→ Immersion in the sanitizing liquid using a container.	→ Complete surface wetting. → Control of the sanitizer concentration. → Does not require closed chambers.	→ Not recommended for dirty eggs. → No control over the applied volume. → Increased difficulty in mass sanitization. → Risk of inducing thermal shock.
Fumigation	→ Release of sanitizing vapors from a sanitizing agent in an enclosed space.	→ Does not require surface wetting. → Control of the sanitizer concentration. → Ease of mass sanitization.	→ Requires hermetically sealed spaces. → Depends on temperature and humidity.
Ultraviolet light (UV)	→ Emission of sanitizing UV from within an enclosed chamber.	→ Does not require surface wetting. → Control of wavelength, intensity, and exposure time.	→ Risk of incomplete surface coverage. → Increased difficulty in mass sanitization. → Requires hermetically sealed spaces.

^1^ Developed with support from Turblin [24], Turtoi and Borda [25], and Oliveira et al. [3].

**Table 2:** In vitro antibacterial activity of ultraviolet light (UV), hydrogen peroxide (H_2_O_2_), peracetic acid (PAA), and ozone (O_3_).

Sanitizer	Exposure Conditions	Sensitive Bacteria	Study
UV	222–254.7 nm; 0.17–60 min	*Acinetobacter baumannii*, *Clostridioides difficile*, *Escherichia coli*, *Klebsiella pneumoniae*, *Listeria monocytogenes*, *Pseudomonas aeruginosa*, *Pseudomonas marginalis*, *Salmonella enterica* serovar Enteritidis, *Staphylococcus aureus*, *Staphylococcus epidermidis*, and *Enterococcus* spp.	[40,41,42,43,44]
H_2_O_2_	0.02–3% or 0.19–1.632 mM/50 μL; 5 min–9 h	*Acinetobacter baumannii*, *Bacillus subtilis*, *Escherichia coli*, *Enterococcus faecalis*, *Enterococcus faecium*, *Enterococcus hirae*, *Klebsiella pneumoniae*, *Pseudomonas aeruginosa*, *Staphylococcus aureus*, *Staphylococcus epidermidis*, and *Salmonella enterica* serovar Choleraesuis.	[45,46,47]
PAA	0.1–0.2%, 6.25–2000 ppm or 0.48–1000 μg/mL; 5–20 min	*Escherichia coli, Proteus mirabilis*, *Pseudomonas aeruginosa*, *Staphylococcus aureus*, *Staphylococcus epidermidis*, *Salmonella enterica* serovar Enteritidis, *Salmonella enterica* serovar Infantis, *Salmonella enterica* serovar Kentucky, and *Salmonella enterica* serovar Typhimurium.	[48,49,50,51,52]
O_3_	0.1–50 ppm or 20–60 μg/mL; 1 min–18 h	*Acinetobacter baumannii*, *Bacillus cereus*, *Bacillus subtilis*, *Escherichia coli*, *Enterococcus faecalis*, *Group A Streptococcus*, *Group B Streptococcus*, *Lactococcus lactis*, *Listeria monocytogenes*, *Pseudomonas aeruginosa*, *Pseudomonas fluorescens*, *Staphylococcus aureus*, *Salmonella enterica* serovar Typhimurium, *Serratia marcescens*, and *Vibrio cholerae.*	[53,54,55,56]

**Table 3:** Effect of ultraviolet light (UV), hydrogen peroxide (H_2_O_2_), peracetic acid (PAA), and ozone (O_3_) on the total bacterial count of hatching eggshells.

Study	Sanitizer	Concentration/Dose	Exposure Time	Application	Total Bacterial Count ^1^
Pereira et al. [60]	UV	254 nm; 0.23 mW/cm^2^	3.5 and 5.5 min	Irradiation	Significant reduction
Pereira et al. [60]	UV	254 nm; 0.23 mW/cm^2^	4.5 min	Irradiation	Non-significant
Clímaco et al. [61]	UV	254 nm; 6.36 mW/cm^2^	1 min	Irradiation	Significant reduction
Melo et al. [22]	UV	254 nm; 8.09 mW/cm^2^	2 min	Irradiation	Significant reduction
Coulibaly et al. [62]	UV	254 nm; 8 W	1 min	Irradiation	Significant reduction
Keita et al. [63]	H_2_O_2_	6%	120 min	Nebulization	Non-significant
Keita et al. [63]	H_2_O_2_	30%	30 min	Vaporization	Significant reduction
Clímaco et al. [61]	H_2_O_2_	1.56%	Not informed	Spraying	Non-significant
Badran et al. [64]	H_2_O_2_	3–7%	Not informed	Spraying	Significant reduction
Melo et al. [22]	H_2_O_2_	3%	Not informed	Spraying	Non-significant
Elbayoumi et al. [65]	H_2_O_2_	5%	Not informed	Spraying	Significant reduction
Wlazlo et al. [66]	H_2_O_2_	30%	Not informed	Spraying	Non-significant
Motola et al. [67]	H_2_O_2_	Not informed	51 min	Spraying	Non-significant
Clímaco et al. [61]	PAA	0.13%	Not informed	Spraying	Non-significant
Melo et al. [22]	PAA	0.3%	Not informed	Spraying	Significant reduction
Motola et al. [67]	PAA	1%	Not informed	Foaming	Significant reduction
Clímaco et al. [61]	O_3_	5–10 ppm	20 min	Fumigation	Non-significant
Melo et al. [22]	O_3_	5–15 ppm	30 min	Fumigation	Non-significant
Wlazlo et al. [66]	O_3_	4.2 mg	5 min	Fumigation	Significant increase
Saad [23]	O_3_	400 mg/s	1–5 min	Immersion	Significant reduction
Dikmen et al. [68]	O_3_	0.05 ppm	1min/h	Fumigation	Significant reduction
Cilavdaroğlu et al. [69]	O_3_	42.8 ppm	6 min	Fumigation	Significant reduction

^1^ When mesophilic counts were not evaluated, we considered the total counts of other bacteria, for example, total *Salmonella* counts.

**Table 4:** Effect of ultraviolet light (UV), hydrogen peroxide (H_2_O_2_), peracetic acid (PAA), and ozone (O_3_) on the total bacterial count of table eggshells.

Study	Sanitizer	Concentration/Dose	Exposure Time	Application	Total Bacterial Count ^1^
Holck et al. [70]	UV	254 nm; 10 mW/cm^2^	0.08 to 5 min	Irradiation	Significant reduction
Mattioli et al. [71]	UV	254 nm	0.25 min	Irradiation	Significant reduction
Rathod et al. [72]	UV	250 nm	1–4 min	Irradiation	Significant reduction
Hasani and Hasani [73]	UV	254 nm; 6.36 mW/cm^2^	1 min	Irradiation	Significant reduction
Knežević et al. [74]	UV	253.7 nm; 10 mW/cm^2^	0.12 min	Irradiation	Significant reduction
Maktabi et al. [75]	H_2_O_2_	0.5%	5 min	Immersion	Significant reduction
Rathod et al. [72]	H_2_O_2_	1.5%	Not informed	Spraying	Significant reduction
Hasani [76]	H_2_O_2_	0.5–3%	Not informed	Spraying	Significant reduction
Vinayananda et al. [77]	PAA	100 mg/kg	2.5 min	Immersion	Significant reduction
Jiang et al. [78]	PAA	0.1%	0.5 min	Spraying	Significant reduction
Neto et al. [79]	PAA	50 ppm	3 min	Immersion	Significant reduction
Neto et al. [79]	PAA	100 ppm	3 min	Immersion	Significant increase
Triginelli [80]	PAA	355 ppm	Not informed	Spraying	Non-significant
Jones et al. [81]	PAA	50–500 ppm	0.17 min	Spraying	Significant reduction
Soares et al. [82]	PAA	50 ppm	1 min	Immersion	Significant increase
Panini [83]	O_3_	1 g/h	1–5 min	Fumigation	Non-significant
Panini [83]	O_3_	1 g/h	10 min	Fumigation	Significant reduction
Yüceer and Caner [84]	O_3_	6 ppm	4 min	Fumigation	Significant reduction
Meireles et al. [85]	O_3_	75–110 ppm	0–70 min	Fumigation	Significant reduction

^1^ When mesophilic counts were not evaluated, we considered the total counts of other bacteria, for example, total *Salmonella* counts.

**Table 5:** Effect of ultraviolet light (UV), hydrogen peroxide (H_2_O_2_), peracetic acid (PAA), and ozone (O_3_) on hatchability.

Study	Sanitizer	Concentration/Dose	Exposure Time	Application	Hatchability ^1^
Pereira et al. [60]	UV	254 nm; 0.23 mW/cm^2^	3.5 and 5.5 min	Irradiation	Non-significant
Melo et al. [22]	UV	254 nm; 8.09 mW/cm^2^	2 min	Irradiation	Non-significant
Coulibaly et al. [62]	UV	254 nm; 8 W	1 min	Irradiation	Non-significant
Souza et al. [101]	UV	254 nm; 0.23 mW/cm^2^	60 min	Irradiation	Significant increase
Keita et al. [63]	H_2_O_2_	6%	120 min	Nebulization	Non-significant
Keita et al. [63]	H_2_O_2_	30%	30 min	Vaporization	Significant increase
Korowiecka et al. [102]	H_2_O_2_	1%	Not informed	Spraying	Non-significant
Al-Shemery and Kamaluddin [103]	H_2_O_2_	0.5–1.5%	Not informed	Not informed	Significant increase
Badran et al. [64]	H_2_O_2_	3%	Not informed	Spraying	Significant reduction
Badran et al. [64]	H_2_O_2_	5%	Not informed	Spraying	Significant reduction
Badran et al. [64]	H_2_O_2_	7%	Not informed	Spraying	Significant reduction
Melo et al. [22]	H_2_O_2_	3%	Not informed	Spraying	Non-significant
Wlazlo et al. [66]	H_2_O_2_	30%	Not informed	Spraying	Non-significant
Tebrun et al. [104]	H_2_O_2_	0.5 mL/m^3^	51 min	Spraying	Non-significant
Pees et al. [105]	H_2_O_2_	0.5 mL/m^3^	51 min	Nebulization	Non-significant
Korowiecka et al. [102]	PAA	1%	Not informed	Spraying	Non-significant
Tebrun et al. [104]	PAA	0.5%	60 min	Foaming	Non-significant
Melo et al. [22]	O_3_	5–15 ppm	30 min	Fumigation	Non-significant
Wlazlo et al. [66]	O_3_	4.2 mg	5 min	Fumigation	Significant reduction
Koc and Aygun [59]	O_3_	1–7 ppm	Not informed	Fumigation	Non-significant
Hrnčár et al. [106]	O_3_	0.45 ppm	Not informed	Fumigation	Significant increase
Saad [23]	O_3_	400 mg/s	1–5 min	Immersion	Significant increase
Dikmen et al. [68]	O_3_	0.05 ppm	1min/h	Fumigation	Non-significant
Cilavdaroğlu et al. [69]	O_3_	42.8 ppm	6 min	Fumigation	Significant reduction

^1^ Compared with the untreated control, but when an untreated control was not available, comparisons were made with formaldehyde or another product used as a positive control.

**Table 6:** Effect of ultraviolet light (UV), hydrogen peroxide (H_2_O_2_), peracetic acid (PAA), and ozone (O_3_) on embryo, chick, and post-hatch performance ^1^.

Study	Observed Effects
	UV
Pereira et al. [60]	UV (254 nm) had no significant effects on embryonic mortality or chick viability. Exposure for 5.5 min was associated with adverse effects on chick quality, characterized by apathy, partially closed eyes, reduced movement, and absence of vocalization. In addition, approximately 20% of the chicks that died in this treatment showed difficulty hatching.
Coulibaly et al. [62]	UV (254 nm) significantly increased embryo length during incubation, relative chick weight at hatch, and the relative weight of the bursa of Fabricius.
Melo et al. [22]	UV (254 nm) had no significant effects on chick quality or yolk sac contamination.
Souza et al. [101]	UV (254 nm) reduced intermediate embryonic mortality and increased late embryonic mortality but did not affect chick weight.
Melo et al. [107]	UV (254 nm) reduced the occurrence of *Staphylococcus*-related genera and decreased the relative frequency of *Enterobacter* in the yolk sacs of newly hatched chicks.
	H_2_O_2_
Keita et al. [63]	H_2_O_2_ (6 and 30%) did not affect chick quality, post-hatch performance, or mortality. Both protocols reduced hatch weight, whereas only the 30% treatment improved the percentage of saleable chicks.
Korowiecka et al. [102]	H_2_O_2_ (1%) had no significant effects on embryonic mortality or malformation rates. It reduced early embryonic mortality (E1–E6) in eggs from 54-week-old breeder flocks.
Melo et al. [22]	H_2_O_2_ (3%) had no significant effects on chick quality or yolk sac contamination.
Badran et al. [64]	H_2_O_2_ (3–7%). The 5% concentration reduced late embryonic mortality, increased chick weight at hatch, increased serum total protein, globulin, glucose, and T3 concentrations, and decreased uric acid, creatinine, AST, and ALT concentrations. The 3% concentration increased late embryonic mortality. The 7% concentration increased intermediate and late embryonic mortality. All concentrations increased total embryonic mortality.
Tebrun et al. [104]	H_2_O_2_ (0.5 mL/m^3^) had no significant effects on embryonic mortality. However, chicks showed lower body weight development during the rearing period.
Wlazlo et al. [66]	H_2_O_2_ (30%) increased early embryonic mortality and reduced late embryonic mortality, without affecting chick weight at hatch or chick survivability during the first 14 days post-hatch.
Pees et al. [105]	H_2_O_2_ (0.5 mL/m^3^) had no significant effects on embryonic mortality, post-hatch mortality, growth performance, or slaughter traits.
Melo et al. [107]	H_2_O_2_ (3%) did not produce specific reported effects on yolk sac microbiota.
	PAA
Melo et al. [22]	PAA (0.3%) had no significant effects on chick quality or yolk sac contamination.
Melo et al. [107]	PAA (0.3%) reduced the occurrence of *Escherichia* spp. and *Staphylococcus*-related genera in the yolk sacs of newly hatched chicks.
Tebrun et al. [104]	PAA (0.5%) had no significant effects on mortality, organ development, or histopathological findings in chicks.
Korowiecka et al. [102]	PAA (1%) had no significant effects on embryonic mortality or malformation rates. It reduced early embryonic mortality (E1–E6) in eggs from 54-week-old breeder flocks.
	O_3_
Melo et al. [22]	O_3_ (5–15 ppm) had no significant effects on chick quality or yolk sac contamination.
Souza et al. [101]	O_3_ (1.6–3.2 mg/L). Both O_3_ concentrations reduced intermediate embryonic mortality while increasing late embryonic mortality. The 1.6 mg/L treatment did not affect chick weight, whereas the 3.2 mg/L treatment reduced chick weight.
Wlazlo et al. [66]	O_3_ (4.2 mg O_3_/h) increased embryonic mortality. Chicks exhibited reduced growth during the first 14 days post-hatch.
Koc and Aygun [59]	O_3_ (1–7 ppm) had no significant effects on embryonic mortality, chick performance, or post-hatch viability.
Dikmen et al. [68]	O_3_ (0.05 ppm) had no significant effects on early, intermediate, late, or pipped embryonic mortality. It reduced chick weight at hatch and yolk sac weight, increased the relative weights of the crop and gizzard, reduced relative heart weight, and decreased body weight and weight gain during the first week post-hatch.
Saad [23]	O_3_ (400 mg/s) significantly reduced total embryonic mortality and improved chick quality. The 2 min exposure produced the highest chick weight at hatch.
Cilavdaroğlu et al. [69]	O_3_ (42.8 ppm) increased embryonic mortality at different stages of incubation.
Melo et al. [107]	O_3_ (5–15 ppm) did not produce specific reported effects on yolk sac microbiota.

^1^ Compared with the untreated control, but when an untreated control was not available, comparisons were made with formaldehyde or another product used as a positive control.

**Table 7:** Comparison of ultraviolet light (UV), hydrogen peroxide (H_2_O_2_), peracetic acid (PAA), and ozone (O_3_) with formaldehyde regarding eggshell bacterial load and hatchability.

Study	Eggshell Bacterial Load (log_10_ CFU/egg or mL)					
	Formaldehyde	UV	H_2_O_2_	PAA	O_3_	S
Pereira et al. [60]	3.92	3.43				ns
Clímaco et al. [61]	1.10 ^c^	2.20 ^b^	3.05 ^a^	2.91 ^a^	2.95 ^a^	*
Melo et al. [22]	1.85	1.64	2.13	1.48	2.33	ns
Coulibaly et al. [62]	<1	<1				ns
Elbayoumi et al. [65]	3.57		3.31			ns
Wlazlo et al. [66]	1.37		1.44		1.54	ns
**Study**	**Hatchability (%)**					
	**Formaldehyde**	**UV**	**H_2_O_2_**	**PAA**	**O_3_**	**S**
Pereira et al. [60]	55.81	70.51				ns
Melo et al. [22]	86.42	87.57	86.37	88.23	86.64	ns
Coulibaly et al. [62]	68.04	72.54				ns
Wlazlo et al. [66]	78.23 ^a^		82.50 ^a^		64.32 ^b^	*

Abbreviation: S, significance; ns, not significant; *, significant. ^a–c^ Different lowercase letters within the same row indicate statistical differences among treatments reported in the respective studies.

## Data Availability

No new data were created or analyzed in this study. Data sharing is not applicable.
